# Subphrenic Abscess as a Complication of Hemodialysis Catheter-Related Infection

**DOI:** 10.1155/2014/502019

**Published:** 2014-07-10

**Authors:** Fernando Caravaca, Victor Burguera, Milagros Fernández-Lucas, José Luis Teruel, Carlos Quereda

**Affiliations:** Department of Nephrology, Hospital Ramón y Cajal, 28034 Madrid, Spain

## Abstract

We describe an unusual case of subphrenic abscess complicating a central venous catheter infection caused by *Pseudomonas aeruginosa* in a 59-year-old woman undergoing hemodialysis. The diagnosis was made through computed tomography, and *Pseudomonas aeruginosa* was isolated from the purulent drainage of the subphrenic abscess, the catheter tip and exit site, and the blood culture samples. A transesophageal echocardiography showed a large tubular thrombus in superior vena cava, extending to the right atrium, but no evidence of endocarditis or other metastatic infectious foci. Catheter removal, percutaneous abscess drainage, anticoagulation, and antibiotics resulted in a favourable outcome.

## 1. Introduction

Infection is a common complication of central venous catheters (CVC) used for vascular access in hemodialysis patients. Gram-positive bacteremia is the typical clinical presentation of CVC related infectious complications.* Pseudomonas aeruginosa *is a less frequent pathogen associated with catheter infection, accounting for 4–16% of isolates [1]. Nevertheless, this pathogen should always be considered as one potential causative agent of CVC related infections, especially in immunocompromised hosts. Metastatic infectious foci are important determinants of the morbidity and mortality of CVC related infections. Endocarditis, septic embolism, and visceral abscesses are rare but serious complications whose mere suspicion demands careful clinical and radiological search.

We report a case of a subphrenic abscess and CVC related bloodstream infection with* Pseudomonas aeruginosa* in a 59-year-old woman on haemodialysis.

## 2. Case Report

A 59-year-old woman with a history of mild intellectual disability and chronic renal allograft dysfunction was admitted to our hospital with a febrile syndrome and a progressive ten-day history of nonproductive cough. She was receiving haemodialysis at a satellite dialysis unit, through a jugular permanent catheter, which had been placed 93 days before. She was not taking corticosteroids or any other immunosuppressive agents, and she had no history of any intra-abdominal disease or recent surgical procedure. The patient complained of intermittent chills and fever up to 39°C for the last few days, with no close temporal relationship with the dialysis session. The rest of anamnesis was anodyne. Physical examination showed a blood pressure of 100/50 mmHg, respiratory rate of 19 breaths/min, and temperature of 38.4°C. Chest auscultation revealed regular heart sounds with a pansystolic murmur, which had already been described in her clinical history, and the breath sounds were absent in the lower third of the right lung. The abdominal exam did not reveal tenderness, hepatomegaly, or masses. Jugular catheter inspection showed inflammatory signs and mild purulent discharge around the exit site.

Her initial WBC was 16 × 10^9^ cells/L (normal range*: *4.5–10.5 × 10^9^ cells*/*L), and she had an absolute neutrophil count of 13 × 10^9^ cells/L. C-reactive protein was 230 mg/L (normal range*: *1–5 mg/dL) and procalcitonin was 1.2 ng/mL (<0.05 ng/mL). No abnormalities were found on the liver function panel. A chest X-ray showed an elevation of the right diaphragm. Cultures from catheter exit site and blood samples were taken, and empiric vancomycin plus gentamicin was prescribed. Forty-eight hours later, the microbiologist reported the isolation of* Pseudomonas aeruginosa *both in exit site and in blood samples. We decided to remove the catheter and to use an arteriovenous fistula, which had been created 64 days before, and it seemed fairly mature. Maki's semiquantitative culture technique of the catheter tip also isolated* Pseudomonas aeruginosa.*

**Figure 1:**
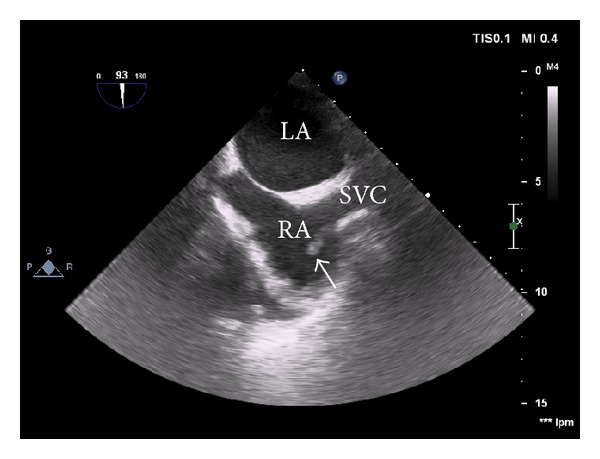
Transesophageal echocardiography showing a tubular 4 × 0.5 cm thrombus (arrow) in superior vena cava (SVC) to the right atrium (RA); left atrium (LA).

A transthoracic and transesophageal echocardiography revealed a tubular 4 × 0.5 cm thrombus in superior vena cava extending to the right atrium, but no signs of endocarditis (Figure 1).

**Figure 2:**
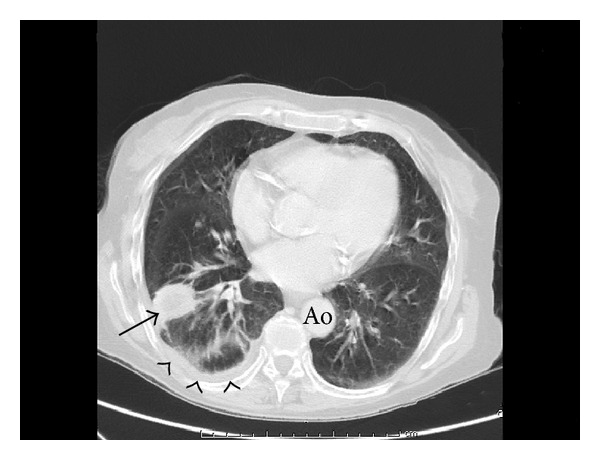
Computed tomography of the chest (lung window) showing a small focal consolidation within the posterior segment of the right lower lobe (arrow) and mild pleural effusion (arrowheads).

**Figure 3:**
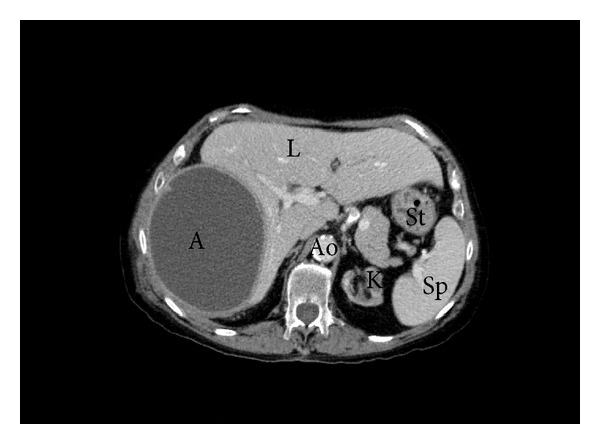
Computed tomography showing a 13 × 10 × 16 cm abscess (A), compressing the liver (L); abdominal aorta (Ao); kidney (K); spleen (Sp); stomach (St).

A computed tomography was done for a better definition of the thrombus, with unexpected findings: a small focal consolidation within the posterior segment of the right lower lobe and mild pleural effusion were described in the lung window (Figure 2), as well as a collection of 13 × 10 × 16 cm in the right hypochondrium and subphrenic space, compressing both the right lung and the liver (Figure 3). The abscess was drained by percutaneous catheter placement, and approximately 800 mL of purulent effluent was obtained in the next few days.* Pseudomonas aeruginosa *was also isolated in the cultures of this purulent drainage.

**Figure 4:**
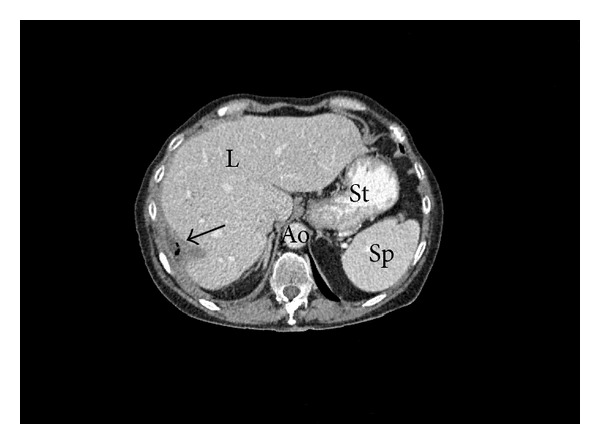
Computed tomography showing a complete healing of the subphrenic abscess (arrow); aorta (Ao); liver (L); spleen (Sp); stomach (St).

According to sensitivity pattern, the patient was treated with oral ciprofloxacin, intravenous tobramycin, and anticoagulation with low molecular weight heparin. The clinical course was favourable, the fever disappeared, and her condition improved rapidly. A transesophageal echocardiogram performed four weeks after admission revealed no thrombus, and another computed tomography confirmed the complete healing of the subphrenic abscess (Figure 4).

## 3. Discussion

To the best of our knowledge, this case described for the first time the association of a subphrenic abscess complicating a central venous catheter infection in a patient on haemodialysis.

Central venous catheters (CVC) have been increasingly used for vascular access in haemodialysis, these devices being the only option in a large percentage of patients [1]. CVC related infectious complications are common and they are associated with high morbidity, mortality, and healthcare costs [2]. The incidence of catheter-related bacteraemia ranges between 0.6 and 6.5 episodes per 1000 catheter-days, being higher in nontunneled catheters compared with tunneled catheters [1]. Gram-positive bacteria are responsible for the majority of CVC related infections in haemodialysis patients.* Staphylococcus aureus* and coagulase-negative* Staphylococcus* are isolated in 50 to 80 percent of these cases. In addition, gram-negative species account for 20 to 40 percent of catheter-related bacteremia [3–6], and* Pseudomonas*/*Stenotrophomonas* species are isolated in 4 to 16 percent of these episodes [1, 7, 8].

*Pseudomonas aeruginosa* is a nonfermentative gram-negative aerobic rod, commonly isolated in the environment, which is responsible for a myriad of infections, especially those of nosocomial origin. This microorganism has a predilection for devitalized tissues and nonbiological materials, such as catheters, prosthesis, and other medical devices [9].

In the case described here, the isolation of* Pseudomonas aeruginosa* in the exit site and tip of the catheter, blood samples, and purulent drainage from the subphrenic abscess suggests a pathogenetic cascade in which the contamination of catheter may have acted as primary source of the infection followed by a haematogenous dissemination, probably with septic emboli formation and, finally, abscess formation in a very unusual location.

Subphrenic abscess formation is a more common complication of intra-abdominal processes (i.e., appendicitis, diverticulitis, etc.) or surgical procedures, although cases of unknown origin have also been described [10]. Isolation of more than one microorganism, especially mixed gram-negative and anaerobic microorganisms, would suggest the intra-abdominal origin of the abscess formation. On the contrary, the isolation of only one microorganism, as in the present case, may rather suggest that the infection was of haematogenous origin.

In our case, the finding of a large thrombus in superior vena cava and right atrium may suggest that the dissemination of the infection could have occurred through pulmonary septic embolisms with abscess formation in the right lower lobe and extension to the subphrenic space by contiguity (subphrenic or hypophrenic empyema).

In this patient, despite bacteraemia and systemic dissemination of* Pseudomonas*, the clinical course was relatively benign without developing septic shock and with a rapid favourable response to antibiotics and abscess drainage. A remarkably good outcome has already been described in* Pseudomonas* bacteraemia associated with CVC in haemodialysis patients [11]. It is likely that prompt catheter removal contributes to the favourable outcome observed in these patients, and, in this regard, the mere fact of* Pseudomonas* isolation and its well-known virulence prevent attempts at catheter salvage, an important risk factor for adverse outcomes after an initial episode of CVC bacteraemia.

The description of this case highlights also the critical importance of a careful clinical and radiological search for metastatic septic foci, especially in those CVC related infections in which a source of septic emboli has been detected (venous thrombus or endocarditis).
